# Behavioural Problems in Children with 46XY Disorders of Sex Development

**DOI:** 10.1155/2017/5987490

**Published:** 2017-06-22

**Authors:** Nalini M. Selveindran, Syed Zulkifli Syed Zakaria, Muhammad Yazid Jalaludin, Rahmah Rasat

**Affiliations:** ^1^Department of Paediatrics, Hospital Putrajaya, Pusat Pentadbiran Putrajaya, Presint 7, 62250 Putrajaya, Malaysia; ^2^Department of Paediatrics, University Kebangsaan Malaysia Medical Centre, Jalan Yaacob Latiff, Bandar Tun Razak Cheras, 56000 Kuala Lumpur, Malaysia; ^3^Department of Paediatrics, Faculty of Medicine, University Malaya, 59100 Kuala Lumpur, Malaysia

## Abstract

The aim of this study is to determine the behavioural problems of children with 46XY disorders of sex development (DSD) with genital ambiguity and to identify the risk factors that may influence behaviour. The 27 participants (aged 6–18 years) consisted of 21 patients raised as boys and 6 patients raised as girls. Control data were obtained from a representative sibling of each patient who was matched for age and gender. The study tool used was the Child Behaviour Checklist (CBCL), which is a parent-administered questionnaire. The analysis of the behavioural scores revealed that the patient group had poorer scores in the total, externalizing, and internalizing realms. This group also had poorer scores in the anxious-depressed, social, and rule-breaking realms as compared to the control group. In addition, the XY-F group had higher scores (more pathological) than the XY-M group, although the difference in the scores was not statistically significant. A comparison of the prevalence of patients with scores in the clinical range with that of the control group was not statistically significant. These findings support the current recommendations that psychological counselling should be an integral part of the professional support offered to patients with DSD.

## 1. Introduction

Disorders of sex development (DSD) are defined as congenital conditions in which the development of chromosomal, gonadal, and anatomic sex is atypical [1]. These disorders are a heterogeneous group of rare conditions. It has been estimated that the incidence of patients presenting with ambiguous genitalia at birth is approximately 1 in 4500–5500 [2, 3]. These individuals are often exposed to numerous diagnostic procedures, including surgery, hormonal treatments, and long-term follow-up, which, in addition to the disorder itself, may cause serious distress [4].

Outcome studies on the psychological well-being of individuals with DSD have tended to focus on the group with congenital adrenal hyperplasia [5–7]. Among those with 46XY DSD, the largest group studied has partial androgen insensitivity syndrome (PAIS) [8]. A long-term follow-up study of adults with PAIS found that there is increased psychological distress in this group, as determined by responses to the Brief Symptom Inventory (BSI) [4]. While it is clear that some individuals with PAIS experience psychological distress, it is as yet unclear which subpopulation is most at risk. Outcomes of mental health in girls and women affected by complete androgen insensitivity syndrome (CAIS) vary greatly across studies. For example, psychological distress, self-harming behaviour, and suicidal tendencies are prevalent in some samples of women with CAIS recruited via physicians or support groups [8]. Similar patterns of psychological distress have been identified from responses to the BSI, and suicidal ideation has been observed in 46XY women with 5-alpha reductase deficiency and 17-beta hydroxyprogesterone deficiency [4, 8].

The management of DSD is complex and has undergone major shifts in the last decade. The initial approach was to apply the “optimal gender policy” whereby the primary thrust of the management was to normalize the physical appearance in line with the gender of rearing [9]. Thus, early constructive surgery was advocated. However, after reviewing reports from patient advocacy groups and clinicians, there are recommendations to postpone any corrective surgery until the child is able to give consent (full consent policy) although not all clinicians agree with this policy [10, 11]. It is thus imperative to assess psychological outcomes of these patients to allow for evaluation of management policies which can then guide future clinical practice guidelines [12, 13].

To this end, in this study, we assessed the behavioural problems of children with 46XY DSD with genital ambiguity. Patients were recruited from the two largest tertiary centres of paediatric endocrinology in Malaysia: University Kebangsaan Malaysia Medical Centre (UKMMC) and University Malaya Medical Centre (UMMC).

## 2. Research Objective

The general objective of this study was to evaluate the behavioural problems of patients with 46XY DSD with genital ambiguity. We also sought to identify any risk factors that may influence behaviour.

## 3. Methods and Study Design

### 3.1. Study Outline

The study was carried out from 1 November 2013 to 30 November 2014. The two centres involved in this study are the major referral centres for this condition in Malaysia as they are equipped with both the paediatric endocrinology and surgical expertise to manage the condition. All patients with 46XY DSD with genital ambiguity aged 6–18 years (*N* = 42) who attended the paediatric endocrine clinic at UKMMC and UMMC were invited to participate in the study. Of those, patients with syndromes, learning disabilities, and neurologic disorders (*N* = 5) were excluded from the study. Patients who did not attend the follow-up despite reminders were also excluded (*N* = 10). The median age of this group was 11.5 (7.8–13.3) years, significantly lower than the patient group. Therefore, a total of 27 patients were included in the study: male social sex *N* = 21 and female social sex *N* = 6. All the controls came from the families of cases (*N* = 27, males = 21, females = 6), and only one sibling from the family closest to the case's age was chosen.

The patients were identified from clinic records. Written informed consent was obtained from the parents (or legal guardians) of all the patients during a clinic visit before participation. After obtaining informed consent, a self-administered questionnaire was distributed to the parents. The questionnaire in all cases was completed by the mother. Approval for the study was granted by the ethical committee of UKMMC and of UMMC. The study protocol conformed to the ethical standards in the Helsinki Declaration II.

Background clinical data were obtained from medical records and included age at diagnosis, karyotype, gender assigned, clinical and biochemical data at presentation including degree of virilization, number and nature of surgical procedures done, and pubertal status. Current information about the patients including pubertal development, complications, medication, and socioeconomic data were obtained by physical examination and interview at the respective clinic.

To group the patients, we applied a classification system based on karyotype and sex of rearing/recent gender as used previously by the German network of disorders of sex development (DSD)/intersexuality and as also suggested by the consensus statement of the ESPE/LWSPE Conference [2, 14] as follows:

- Subgroup 1 (XY-F, *N* = 6): individuals with XY karyotype and reared as girls/living as women
- Subgroup 2 (XY-M, *N* = 21): individuals with XY karyotype and reared as boys/living as men.

### 3.2. Questionnaire

The study tool was the Child Behaviour Checklist (CBCL). The proxy version of this tool was used, that is, the parents answered the questionnaire. An English version and a version translated into Bahasa Malaysia (BM) (the national language of Malaysia) were used. The latter has been validated according to international translation standards with separate backward and forward translations [15, 16].

The CBCL used in this study consisted of 118 items or questions. The parents answered the questions based on the child's behaviour over a 6 month period. Parents rated their child's demonstration of behaviours by scoring 0 for never, 1 for sometimes, and 2 for always. The behavioural problem scales are derived from various combinations of the items or questions. The questionnaire provides three summary scales and eight syndrome scales. The information from the questionnaires was entered into a CBCL software program, which calculated the raw scores, *T* scores, and score percentiles. The *T* scores of the CBCL summary and syndrome scales were used in the analysis. A high *T* score indicates a higher risk of psychopathology in a particular behavioural scale. The *T* scores of the summary scales that were above 63 (>97th percentile of the normative sample) and those of the syndrome scales that were above 69 (>97th percentile of the normative sample) were considered to be the clinical range scores.

### 3.3. Statistical Analysis

Data was analysed using IBM SPSS version 22. Statistical difference was assessed by using the *t*-test for parametric data and the Mann–Whitney/Kruskal-Wallis tests for nonparametric data. For categorical data, the *X*^2^ test was used. For identification of the risk factors, bivariate analysis (Pearson's correlation) was performed. Statistical significance was assumed when *P* < 0.05. However, for scale scores, this was adjusted using the Bonferroni-Holm method to account for multiple comparisons.

## 4. Results

### 4.1. Patient Cohort and Control

There was no significant difference between the patient and control groups with regard to age at CBCL administration, gender distribution, mean family income, and parental marital status (Table 1). Similarly, there was no difference between male and female patients as compared to male and female controls with regard to demographic characteristics.

Patient characteristics are summarized in Tables 1, 2, and 3. The median age of the population was 14 years (12–15). Twenty-one patients were included in the group XY-M with a median age of 14 years (10–15), and 6 patients were included in the group XY-F with a median age of 14.5 years (12.8–18). There was no statistical significance between the ages of the two groups. Eighteen patients (66.7%) were adolescents (above 12 years old).

The majority of patients had already had some surgical procedure, with 14 patients having undergone a genital reconstruction. Eight patients had three or more surgical procedures. Nine patients had had a gonadectomy, in seven cases bilateral. Two patients had a gonadoblastoma at the time of removal of gonads. One patient with ovotesticular DSD underwent gender change from female to male.

### 4.2. CBCL Scores

The total, externalizing, and internalizing scores were significantly higher (more pathological) in the patient group as compared to the control group (Table 4). Analysis of syndromic scale scores revealed that the patient group had significantly poorer scores in the anxious-depressed, social, and rule-breaking dimensions (Table 5). The XY-F group had higher scores (more pathological) than the XY-M groups, although the difference was not statistically significant (Table 6). However, the numbers in each group were small, thereby limiting the statistical power.

### 4.3. Prevalence of DSD Patients with Clinical Range Scores

A chi-square test of independence was performed to examine the proportion of DSD cases with clinical range scores as compared to the controls. The analysis revealed that the difference between the two groups in terms of prevalence of clinical range scores was not statistically significant (Table 7).

### 4.4. Associations

A Spearman's rank-order correlation was run to determine the relationship between family income and the CBCL scores. The results showed that there was no significant correlation between family income and the total, internal, and external CBCL scores. There was also no significant correlation between degree of virilization (Prader score) at diagnosis and the total CBCL scores for the population XY-F (*r* = −0.62, *P* = 0.19) and XY-M (*r* = 0.12, *P* = 0.61). In addition, an evaluation of the effect of repeated surgeries on the total, internal, and external CBCL scores did not reveal a significant association. To determine the effect of the mode of puberty on behavioural outcomes, the total, internalizing, and externalizing scores of the group who had experienced spontaneous puberty were compared with those of the group who had undergone induction of puberty. There were 4 patients who went into spontaneous puberty, whereas 17 patients had pubertal induction. The group of patients who went into spontaneous puberty had a median age of 14 years (13.3–14), whereas the group of patients whose puberty was induced had a median age of 15 years (13–17). There was no significant difference between the groups.

## 5. Discussion

In this study, we found that the patient group experienced increased behavioural problems as compared to the control group. The analysis of the behavioural scores revealed that the patient group had significantly poorer scores in the total, externalizing, and internalizing realms. This group also had significantly poorer scores in the anxious-depressed, social, and rule-breaking realms as compared to the control group. This finding is in line with the existing literature. Zhu et al. [17] demonstrated that in a group of patients with DSD, psychological problems were significantly more prevalent in the DSD group than in the control group. However, the prevalence of patients with scores in the clinical range as compared to the control group did not attain statistical significance. It is possible that we did not find evidence of severe emotional problems because of the “sleeper effect,” whereby patients struggle later in adulthood when the implication of having DSD becomes more real [18].

Patients with DSD are at greater risk of behavioural problems and poor psychological outcomes because they encounter development hurdles that are intense and that are likely to have negative psychological implications [19]. Repeated surgery and medical intervention may also have a negative influence on the psychological outcome [20]. However, in this study, we were unable to demonstrate an association between behavioural outcomes and surgery. The complexity of the condition may also create psychological distress in patients. This anxiety may impair cognitive processing and further impair understanding of the condition. Furthermore, although the consensus in the field is to recommend early disclosure to patients, in our experience, most parents are reluctant to divulge the diagnosis to their children. They feel that this may deprive their children of a happy childhood. However, there is evidence to the contrary that patients who are well informed have a greater chance of developing coping skills and adjusting to the limitations that their diagnosis may impose on them [21]. Conversely, in a culture where a diagnosis of DSD is considered shameful, the added burden of social isolation and stigmatization may contribute to increasing psychological distress [22, 23].

We hypothesized that the greatest degree of psychological distress would be found in the groups where there was a discrepancy between the genetic and social sex [24]. Jürgensen et al. [25] in a study of 166 children with DSD aged 4–12 years demonstrated that girls with DSD and androgen effects show increased cross-gender tendencies. The authors postulated that this may be either as a direct consequence of androgen effects or as a more indirect consequence of a feeling of “being different” caused by atypical physical and/or behavioural characteristics [25]. Thyen et al. [9] in a study of 110 adults with DSD demonstrated that the XY-F group reports the lowest scores of satisfaction with the care received [9]. Our study did demonstrate a trend of poorer scores in the XY-F group, although it was not statistically significant. The rarity and complexity of this group often leads to a delay in diagnosis and also to a delay in accessing specialized medical care. Thus, this may be a group that needs greater surveillance and psychological support. The authors of a German clinical evaluation study [25] also recommend that this group (girls with androgen effects) receive greater attention as there is a risk of gender insecurity and dysphoria. However, they also conclude that gender of rearing and socialization also play a role in gender identity development [25]. Thus, for this group of patients, parenting and social relationships are of paramount importance and the patients and their families should receive appropriate support throughout the patient's development phase.

Our study does have some limitations. Firstly, the questionnaire used was not specifically designed for the DSD conditions assessed. However, we do believe that the questionnaire addressed most of the domains that would be affected in patients with sex disorders. The results of this study are also derived from a survey completed by the participants' parents. It is likely that there would be some discrepancy between the results gained from the parents and from the patients themselves. Therefore, future research will need to evaluate the patient's perception as the primary stakeholder. Also, the control group did not include individuals with a chronic medical condition. Presence of a chronic medical condition has been demonstrated to have an impact on self-esteem, body image, and risk of depression [26]. We also used the Prader scores to evaluate the degree of virilization. As the score relates primarily to virilization of the female genitalia in cases of congenital adrenal hyperplasia, the application of this score to other conditions may be inaccurate. Due to the rarity of the disorders, we were also hampered by a small sample size. This also prevented us from undertaking a disorder-specific analysis.

For a comprehensive analysis of the psychological outcomes of this group of patients, we will also need to take into account assessment of body image, self-image, and social relationships. This will be a challenging task given the reluctance of the current culture in Malaysia to open up and talk about these issues. Researchers will have to find ways to overcome the subjects' reticent in divulging information on these issues [19]. We also acknowledge that the psychological outcomes of a child with DSD are heavily influenced by family dynamics and parenting. Thus, in the future, we will also need to explore the link between parental outcomes and the patient's psychological outcomes to assess the degree to which the former influences the latter. This will also aid in the identification of families who are at risk. Studies have also demonstrated that the DSD patient's outcome is also influenced by the medical practitioner's attitude and also willingness to explore psychosocial aspects of disorder management [27]. We will need to evaluate this in future studies to determine the extent to which this influences the patient's psychological outcome.

In summary, this study demonstrated that children and adolescents with DSD have a higher degree of psychological distress. These findings support the current recommendations that psychological counselling should be an integral part of the professional support offered to patients with DSD [28–30]. Application of evidence-based psychological interventions will be able to improve the quality of care received by these patients. The findings reported here also underline the importance of establishing paediatric mental health services to support the social and behavioural development of these patients.

## Figures and Tables

**Table 1:** Sociodemographic characteristics of cases and control.

Variable	Case	Control	*P* value
*Median age at study entry in years (IQR)* ^a^	14 (3)	13 (3)	0.37
*Gender frequency (%)* ^b^			0.75
Male	21 (77.8)	20 (74.1)	
Female	6 (22.2)	7 (25.9)	
*Mean family income * *RM (SD)* ^c^	3851 (1343)	4000 (1487)	0.70
*Parental marital status* ^d^			
Married	26	26	1.00
Single	1	1	

^a^Mann–Whitney; ^b^chi-squared test; ^c^independent *t*-test; ^d^Fisher's exact test.

**Table 2:** Baseline characteristics of DSD cases.

	Male (*N* = 21)	Female (*N* = 6)
Age in years at study entry^a^	12.6 (3.6)	15 (2.7)
Prader score^b^		
2		2 (28)
3	2 (9)	4 (72)
4	13 (65)	
5	6 (26)	
Surgical procedures^b^	16 (76.2)	5 (83.3)
Number of surgical procedures		
≤2	8	4
>2	8	1
Gonadectomy	5	4

^a^Mean (standard deviation); ^b^frequency (%).

**Table 3:** Chart showing the grouping of patients and the distribution of diagnosis.

		Group		Total
		XY-M	XY-F	
Diagnosis	Gonadal dysgenesis	13	0	13
	Mixed gonadal dysgenesis	3	1	4
	Androgen insensitivity	1	5	6
	Ovotestis	3	0	3
	5-alpha reductase deficiency	1	0	1
Total		21	6	27

**Table 4:** Total, internalizing, and externalizing *T* scores from the CBCL in patients with XY DSD as compared to the control group.

	Case	Control	*P* value
Internalizing score^∗^	52 (19)	39 (17)	0.002
Externalizing score^∗^	51 (17)	40 (16)	0.020
Total^∗^	50 (27)	36 (16)	0.025

∗ denotes significant variables. Scores are given as median (IQR) with higher scores denoting poorer outcome.

**Table 5:** Syndrome scale *T* scores in patients with XY DSD as compared to the control group.

	Case	Control	*P* value
Anxious-depressed^∗^	53 (8)	50 (1)	0.004
Withdrawn-depressed	53 (13)	50 (0)	0.007
Somatic problems	50 (8)	50 (3)	0.287
Social^∗^	55 (13)	50 (3)	0.002
Thought	50 (5)	50 (0)	0.009
Attention	53 (10)	50 (1)	0.039
Rule-breaking^∗^	53 (10)	50 (1)	0.005
Aggressive	51 (7)	50 (1)	0.096

∗ denotes significant variables. Scores are given as median (IQR) with higher scores denoting poorer outcome. *P* value was adjusted using the Bonferroni-Holm method to account for multiple comparisons.

**Table 6:** Total, internalizing, and externalizing *T* scores from the CBCL in patients with XY-M and XY-F.

	XY-M	XY-F	*P* value
Internalizing score	52 (16)	52.5 (32)	0.76
Externalizing score	50 (13)	60 (28)	0.24
Total	49 (17)	57.5 (33)	0.32

Scores are given as median (IQR) with higher scores denoting poorer outcome.

**Table 7:** Percentage of XY DSD patients in clinical range on syndromic scores as compared to controls.

	Case (*N*)	Control (*N*)	*P* value^a^
Internal	22.2% (6)	3.8% (1)	0.10
External	3.7% (1)	7.4% (2)	1.0
Total	13.3% (4)	0%	0.11

^a^Fisher's exact test.
